# Leveraging Multi-Tissue, Single-Cell Atlases as Tools to Elucidate Shared Mechanisms of Immune-Mediated Inflammatory Diseases

**DOI:** 10.3390/biomedicines12061297

**Published:** 2024-06-12

**Authors:** Anthony K. McLean, Gary Reynolds, Arthur G. Pratt

**Affiliations:** 1 Translational and Clinical Research Institute, Newcastle University, Newcastle upon Tyne NE2 4HH, UK; 2 Center for Immunology and Inflammatory Diseases, Massachusetts General Hospital, Boston, MA 02114, USA; 3 Musculoskeletal Unit, Newcastle upon Tyne Hospitals NHS Foundation Trust, Newcastle upon Tyne NE7 7DN, UK

**Keywords:** immune-mediated inflammatory diseases, single-cell sequencing, multi-tissue atlas, cross-tissue atlas, human cell atlas, integrative analysis, precision medicine

## Abstract

The observation that certain therapeutic strategies for targeting inflammation benefit patients with distinct immune-mediated inflammatory diseases (IMIDs) is exemplified by the success of TNF blockade in conditions including rheumatoid arthritis, ulcerative colitis, and skin psoriasis, albeit only for subsets of individuals with each condition. This suggests intersecting “nodes” in inflammatory networks at a molecular and cellular level may drive and/or maintain IMIDs, being “shared” between traditionally distinct diagnoses without mapping neatly to a single clinical phenotype. In line with this proposition, integrative tumour tissue analyses in oncology have highlighted novel cell states acting across diverse cancers, with important implications for precision medicine. Drawing upon advances in the oncology field, this narrative review will first summarise learnings from the Human Cell Atlas in health as a platform for interrogating IMID tissues. It will then review cross-disease studies to date that inform this endeavour before considering future directions in the field.

## 1. Introduction

Immune-mediated inflammatory diseases (IMIDs) are a group of conditions characterised by immune dysfunction leading to chronic inflammation and tissue damage [1]. IMIDs can affect various organs, including the joints (e.g., rheumatoid arthritis; RA), gut (inflammatory bowel disease; IBD), and skin (e.g., psoriasis, atopic dermatitis). It is known that people with one IMID are more likely to develop another compared to the general population. For example, the incidence of IBD is 2.5 times higher amongst people with RA compared to the rest of the UK population [2]. Over the last two decades, IMID incidence has increased significantly [2]. In parallel, evolving pathophysiological understanding of these conditions coupled with advances in biotechnology have led to the deployment of targeted, biologic immunotherapies that have transformed patient outcomes. This era was heralded by TNF inhibitors (TNFis) for the treatment of RA [3], and the drug class has since proved effective for several other IMIDs, including psoriasis, psoriatic arthritis (PsA), axial spondyloarthritis (AxSpA) and IBD (Crohn’s disease and ulcerative colitis; CD and UC). However, it has also become evident that a given targeted approach typically benefits only a subset of patients with each IMID for which it is licensed. Hence, around half of AxSpA or IBD recipients of TNFi achieve satisfactory outcomes [4,5,6]; alternative targeted therapies may prove effective for the remainder, but, in the absence of suitable predictive biomarkers, such patients remain subject to “trial-and-error” treatment approaches. On one hand, such variation in therapeutic efficacy between individuals with the same diagnosis highlights heterogeneity in the molecular, cellular, and immunopathological drivers of each clinically classified IMID [7]. On the other hand, the pleiotropic benefit of targeted treatments between IMIDs recalls overlapping genetic architectures that point to shared disease mechanisms [8]. It follows that an organ-specific classification of IMIDs is limited in addressing patient need, as it fails to acknowledge endotypes that may exist within and between IMIDs. A complimentary classification based on tissue-based pathology could prove transformative, enabling the right drug to be selected for the right patient.

As with other fields, our understanding of immunobiology has been revolutionised by next-generation sequencing (NGS). Bulk RNA-sequencing measures average gene expression levels over a large population of cells, which may be composed of different cell types. Although bulk profiling enables a gross comparison of conditions (e.g., healthy versus diseased states), it cannot differentiate between changes in cell type abundance and cell type-specific gene expression [9]. Deconvolution methods enable cell type proportions to be estimated from such data, but their accuracy is limited. Advancements in single-cell technologies have allowed for the profiling of tens of thousands of individual cells in parallel. Unlike bulk RNA-seq, single-cell RNA-seq enables the interrogation of tissue/organ composition, heterogeneity of cell states, and rare cell populations; they have proven pivotal in describing molecular and cellular heterogeneity at the level of blood and tissue. Despite their capabilities, such approaches rely on tissue dissociation, meaning that the spatial context of the cells is lost. Spatial omics technologies can map measurements of biomolecules in tissue sections, in some cases up to single-cell resolution. These advances have been complemented by the rapid development of algorithms and standardised pipelines for data analysis. Myriad computational tools have become available to facilitate alignment, quality control (QC), quantification, dimensionality reduction, batch correction, and clustering of single-cell sequencing (scSeq) data. Toolkits such as Seurat [10] and Scanpy [11] combine many of these functions into integrated workflows, the latter utilising a Python-based framework for better scalability and integration with machine learning applications. Taken together, evolving technologies of this kind and bioinformatics tools have the ability to dissect the heterogeneity within IMID tissues at unprecedented depth and scale.

These advances, combined with the growing availability of IMID datasets and associated metadata, provide an opportunity to deliver a step-change in the characterisation of shared and tissue discrete pathobiology. In this review, an overview of cross-tissue analyses of health will be given to explore the challenges of multi-tissue atlas construction. Next, pan-cancer atlases will be explored as an exemplar for the characterisation of shared cell states in disease and their association with clinical outcomes. Finally, current multi-tissue studies of IMIDs will be reviewed, and future directions will be explored.

## 2. Lessons from the Human Cell Atlas (HCA)

Significant advancements in single-cell genomics have enabled the construction of single-cell “atlases” of tissues that make up the human body. Founded in 2016, the Human Cell Atlas (HCA) is a global consortium that aims to develop comprehensible reference maps of tissues for understanding health and disease [12]. This effort will produce draft atlases of increasing resolution and scale. The first will feature single-cell and single-nucleus transcriptomic data from healthy tissues, combined with spatial analysis. HCA researchers have profiled 58.5 million cells across 15 organ systems to date [13]. Commensurate with their objectives; however, the HCA’s Biological Network atlases map discrete tissues, organ systems, or focus areas such that they, with the limited exceptions described here, do not yield cross-tissue single-cell atlases. 

### 2.1. Cross-Tissue Studies in Health

In 2022, four studies detailing multi-tissue single-cell atlases were published in Science [14,15,16,17]. Collectively, they profiled over 1 million cells, corresponding to 500 cell types across more than 30 tissues. Domínguez Conde et al. [14] constructed a multi-tissue immune cell atlas including ~330,000 immune cells across 16 tissues from 12 deceased adult donors. This involved 10*X* scRNA-seq and paired VDJ sequencing of T and B cell receptors. These authors furthermore developed a machine learning tool named CellTypist that can utilise manually curated, harmonised cell type labels for automated annotation of scRNA-seq data. Suo et al. [15] constructed a multi-tissue atlas of the developing immune system, also using scRNA-seq and paired VDJ sequencing. This atlas featured >900,000 cells across nine prenatal tissues, including the yolk sac, liver, and skin. Eraslan et al. [16] exploited single-nucleus RNA sequencing (snRNA-seq) to construct a multi-tissue atlas featuring >200,000 cells from frozen, archived tissue samples across 8 healthy organs. Finally, the Tabula Sapiens Consortium [17] created a multi-tissue atlas of ~500,000 cells from 24 different tissues and organs using 10*X* scRNA-seq and SMART-seq2 technologies. Collectively, these landmark studies demonstrate the potential to delineate shared and tissue-discrete cell states. For example, Eraslan et al. [16] identified a shared fibroblast phenotype of extracellular matrix protein expression across multiple tissues and a distinct calcium signalling programme in lung alveolar fibroblasts. Domínguez Conde et al. [14] found tissue-discrete immune cell subsets with variable chemokine expression, indicative of adaptation to tissue microenvironments. These studies also allowed for the identification of rare cell populations, such as enteric neurons in the oesophagus and prostate neuroendocrine cells [16]. Finally, these studies reported enrichment of disease-associated loci in specific cell types/states. Ultimately, the HCA multi-tissue atlases demonstrate how cross-tissue comparison and association of disease with specific cell types can deliver valuable insights into human physiology. 

### 2.2. Cross-Tissue Studies in Health: Challenges and Potential Solutions 

HCA Consortium studies such as these provide insight into the challenges of single-cell, multi-tissue atlas construction and interrogation, but also valuable precedent and some approaches for addressing them. These are summarised below.

#### 2.2.1. Methods for Sample Processing

The construction of large-scale atlases is dependent upon the availability of viable single-cell suspensions of freshly isolated tissues and, in turn, robust translational research infrastructure linking clinical and laboratory facilities/collaborating teams. Disaggregation protocols for these purposes have been validated for many tissues [18], but it is important to note that these may not be suitable for certain target cell types that, due to their morphology, do not readily form single-cell suspensions. For example, neurons form complex networks via their branched extensions (axons and dendrites), epithelial cells are sensitive to most digestion protocols, and viable yield is often poor, while skeletal myocytes and cardiomyocytes are multinucleated and large in size. Enzymatic or mechanical dissociation of these tissues can disrupt cellular integrity, bias cell populations, and introduce stress response artefacts. Single nuclear (sn)RNA-seq circumvents some of these challenges and is also applicable to frozen, archived tissue samples. Four nucleus isolation protocols for doing so have now been benchmarked by Eraslan et al. [16]. Such work broadens opportunities for snRNA-seq of frozen tissues at a much larger scale than could previously be contemplated, for example, by facilitating studies to elucidate mechanisms of genetic risk by identifying cell types across tissues via which expression quantitative traits (eQTLs) are exerted at disease risk loci. 

It is not practical or desirable for a single laboratory to generate all the data that constitutes a cell atlas. However, data production is not standardised across centres, which inevitably introduces “batch” effects that must be distinguished from biology. The broad concept of “batch” includes all non-biological factors that contribute to variability, including sample processing, scSeq platform technology, sequencing, reference genomes, and alignment tools. The unwanted variation introduced by these factors can result in significant difficulties when making cross-study comparisons.

#### 2.2.2. Challenges of Variation between scSeq Platforms

The scSeq platform used to generate scRNA-seq data can be a significant source of variation. Most studies use the droplet-based 10*X* Genomics platform [19]. However, as single-cell technologies have matured, versions of the same technology can differ in terms of sequencing end (3′ versus 5′) and droplet capture (Next GEM versus GEM-X [20]), thus causing further variation. Sequencing reads must be aligned to a reference genome once they have been generated, which introduces additional sources of variation, such as whether intronic reads are included and the choice of reference (UCSC [21] versus Ensembl [22]) and its version number. Differences in alignment methods can be mitigated by realignment where the original FASTQ files are available, but, as discussed below, the availability of these files is a significant barrier to data reuse and high-quality atlas generation. 

#### 2.2.3. Challenges of Data Access

Aside from pooling archived tissues, an alternative method of increasing sample size for atlas construction is to leverage publicly available datasets. Public data repositories such as the HCA Data Portal [12], the European Genome-phenome archive (EGA) [23], and the Gene Expression Omnibus (GEO) [24] have sought to “democratise” access to scRNA-seq datasets. Processed data are frequently stored on GEO, but to standardise all stages of data processing (e.g., alignment, QC, normalisation, etc.), raw data in the form of FASTQ files are required. Due to concerns pertaining to donor identification, access to raw data requires the submission of data access agreements. Multiple repositories exist, but access processes differ between them, making it challenging for single-centre administrators to navigate multiple agreements. Even more problematically, raw data are frequently not available at all or only through requests to the author.

#### 2.2.4. Methods for Data Integration 

As their availability, scale, and complexity have increased, data integration has become a key component of computational analysis pipelines. Indeed, Suo et al. [15] demonstrated this by integrating newly generated scRNA-seq data from the prenatal yolk sac, spleen, and skin with publicly available single-cell foetal datasets. Just as samples handled in batches during processing and data generation lead to unwanted technical variation within datasets as a result of differences in sequencing depth or read length, “between-dataset” batch effects are an almost inevitable consequence of combining data from different studies. It is critical to minimise technical variation while preserving biological variability for downstream analyses. Numerous integration methods are available for scRNA-seq data, which may be variously suited to this objective from study to study (Table 1). “Similarity-based methods” generally project cells into a low-dimensional embedding, identify similar cells/clusters across batches and then apply batch correction at these levels. Some similarity-based methods do not apply batch correction, instead producing a batch-weighted graph. “Deep learning methods” utilise variational autoencoder (VAE) frameworks to learn a latent representation of cells and then decode this to infer batch-corrected estimated counts.

#### 2.2.5. Methods for Benchmarking Data Integration 

Benchmarking of data integration methods used to solely rely on qualitative evaluation of UMAP visualisations, which involves users observing the degree of batch mixing and the separation of cell type labels from 2D plots. While this approach is informative, it is also highly subjective. Now, packages such as scIB (single-cell integration benchmarking) provide a platform for quantitative evaluation of scRNA-seq using metrics of “batch mixing” and “biological conservation” (Table 2) [27]. Batch mixing refers to the combination of data from different batches via the removal of unwanted technical and biological variation. Conversely, biological conservation refers to the preservation of relevant biological variability between cells, which is commonly captured by cell type annotations.

Importantly, these performance metrics complement, rather than replace, the inspection of UMAP visualisation. Benchmarking studies have shown that there is no integration method that is universally superior, so users are advised to conduct benchmarking on their own data to select the most appropriate method [34]. This is due to a lack of “ground truth” in integration benchmarking: the “true” batch-corrected structure of a dataset is unknown, and the preservation of user-defined cell type labels is used to measure of bio-conservation. Consequently, the choice of integration method is often dependent on researcher preference and familiarity. Interestingly, all four of the HCA multi-tissue atlases [14,15,16,17] used VAE-based integration methods to integrate their datasets. This preference for VAE-based integration methods in these studies can be partly attributed to recommendations from benchmarking studies on complex batch effects [27]. 

#### 2.2.6. Methods for Cell Type/State Annotation 

Along with integration, cell type/state annotation remains a challenge in single-cell transcriptomics. The ‘traditional’ strategy involves manual annotation using curated lists of marker genes. These marker genes are then compared with genes that are differentially expressed between cell clusters to annotate cell types. This approach is intuitive and based on scientific consensus; however, it is also time-consuming and lacks reproducibility. Consequently, several methods for automatic cell-type annotation of scRNA-seq data have arisen over the last five years (Table 3). “Marker-based” annotation methods score and classify cells based on their expression of cell-type-specific marker gene sets. “Reference-based” annotation methods transfer cell type labels from a reference to cells or clusters in a query dataset with similar gene expression profiles. This can be achieved by several approaches, including correlation, supervised learning, and reference mapping. Supervised learning methods, such as the aforementioned CellTypist tool [14], involve training classifiers on labelled reference datasets and propagating cell type labels onto an unlabelled query dataset. In contrast, reference mapping approaches involve projection of the query dataset into the same low-dimensional space as the reference and subsequent label transfer using this joint embedding.

HCA efforts aim to build a definitive reference atlas of all cell states with a harmonised and uniform annotation. In the future, it will be important for researchers to be able to annotate their data using this curated reference. This will aid consistency across studies and reduce time spent on manual annotation. For most HCA tissues, only small studies, exist and there is no coherent annotation across them, meaning that the current focus remains on the discovery of cell states. Nevertheless, reference-based automated annotation tools are likely to be very important in the future when the HCA goals of a unified cell type nomenclature are realised.

## 3. Learnings from Pan-Cancer Studies

**Figure 1:**
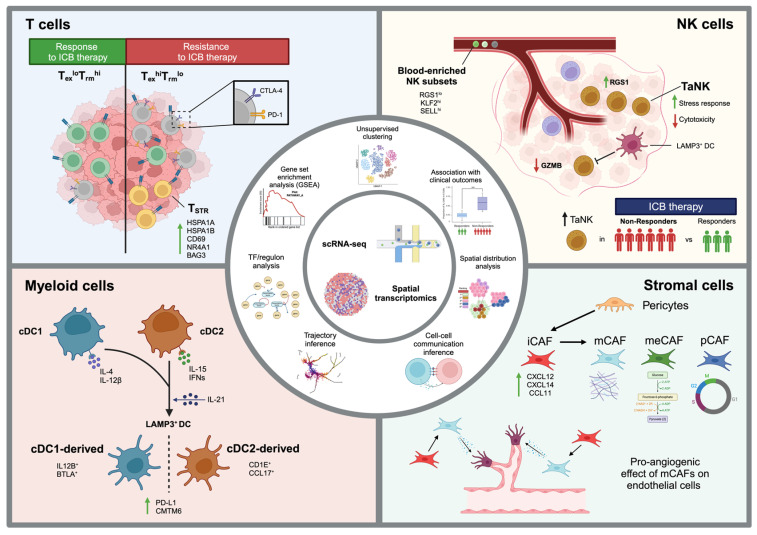
Learnings from Pan-cancer atlases. Summary of technologies, analyses and findings from pan-cancer, single-cell atlases of T cells [62,63], NK cells [64], myeloid cells [66], and stromal cells [67]. Green arrows (↑): upregulation; Red arrows (↓): downregulation. Myeloid cells” section of Figure 1 was reproduced from Cell, Vol 184, Cheng et al., A pan-cancer single-cell transcriptional atlas of tumour infiltrating myeloid cells, 792–809, Copyright (2021), with permission from Elsevier. Created with BioRender.com.

When translating learnings from cross-tissue atlases in health that are of relevance for IMID tissue comparisons using scSeq data, reference to the oncology field is instructive, where analogous evaluations between tumour tissues already form part of the established literature (Figure 1). For example, a pan-cancer T cell atlas (~400,000 cells, 316 donors, 21 cancer types) developed by Zheng et al. [62] included newly generated and publicly available scRNA-seq data integrated using Harmony (Table 1) [34]. Two major immunophenotypes emerged, defined according to relative frequencies of terminally exhausted and tissue-resident memory CD8^+^ T (T_ex_ and T_rm_) cells infiltrating tumour tissue—the relative ratios of which appear to discriminate between good and poor cancer outcomes. Another pan-cancer T cell atlas developed by Chu et al. [63] (~300,000 cells, 324 donors, 16 cancer types) [39] identified CD4^+^ and CD8^+^ T cells displaying a stress response phenotype (T_STR_ cells), which mapped to lymphocyte aggregates near tumour beds across multiple tumour types. Of particular note, in a subset of immune checkpoint blockade (ICB) recipients with renal cell carcinoma and melanoma, high CD4/CD8^+^ T_STR_ frequencies were associated with poor cancer responses.

Tang et al. [64] produced a natural killer (NK) cell atlas (~160,000 cells, 716 donors, 24 cancer types). Distinct infiltrating NK cell subsets displayed cancer type specificity, with CX3CR1^+^ NK cells associated with pancreatic cancer, breast cancer, and melanoma. Regulator of G-protein signalling 1 (RGS1) was identified as a potential marker of tumour-infiltrating NK cells, due to its high specificity amongst differentially expressed genes between blood and tumours. Stressed CD56^dim^CD16^hi^ NK cells were also specifically enriched in tumours compared to blood, thereby termed tumour-associated NK (TaNK) cells, which were found to exhibit impaired cytotoxicity compared to NK cell subsets in adjacent non-tumour tissues, with lower granzyme B (GZMB) and perforin levels measured by multiplex immunofluorescence (mIF) staining and flow cytometry. A high abundance of TaNK cells in breast cancer and melanoma tumours prior to ICB therapy was proposed as a predictor of non-response to therapy. The authors furthermore leveraged tools, including CellPhoneDB [65], together with previously annotated pan-cancer atlases of T cells and myeloid cells [62,66], to infer an immunosuppressive role for TaNK cells in the tumour micro-environment via interaction with other immune cell compartments. 

Spatial transcriptomics (ST) has proved a valuable adjunctive tool alongside scSeq analyses of disaggregated tumour tissue for purposes of unravelling pathobiology within and between tumour types. A study by Ma et al. [67] exemplifies this, whereby a pan-cancer spatial atlas of fibroblasts, pericytes, and smooth muscle cells (SMCs) was developed by integrating scRNA-seq and ST data (~740,000 cells, 6 cancer types, 56 donors for scRNA-seq, 22 donors for ST). CellTrek, a computational tool that uses scRNA-seq and ST data to map cells back to their spatial coordinates in tissue sections, was used for this purpose [46]. Distinct from ST deconvolution methods, which infer cell type proportions for each coordinate, this approach helped to characterise four cancer-associated fibroblast (CAF) subtypes: inflammatory (iCAF), matrix (mCAF), metabolic (meCAF), and proliferative (pCAF); iCAFs displayed enrichment of chemokine and complement activation genes, while mCAFs displayed enrichment of those related to angiogenesis and regulation of extracellular matrix (ECM) organisation. A transition pathway from pericytes to iCAFs and mCAFs was also proposed by applying the Slingshot tool [68]. 

All of these studies illustrate the enormous potential for comparative, cross-tissue scSeq analyses. Incorporating innovative analytical tools and/or in combination with parallel technologies, including spatial transcriptomics/proteomics of paired samples, such approaches may unravel discrete and overlapping mechanisms of disease across different tumours. As will now be highlighted, deploying them across tissue from distinct IMIDs may offer similarly valuable insight, though the field remains in its infancy.

## 4. Cross-Tissue Studies of IMIDs

**Figure 2:**
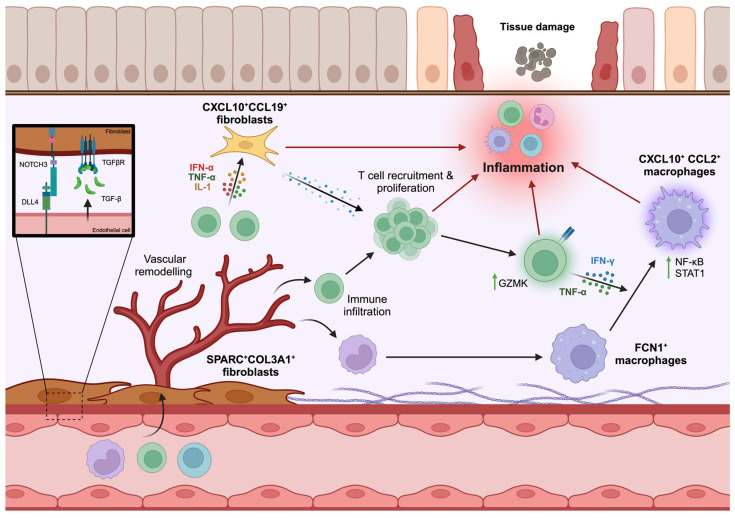
Cross-tissue studies of IMIDs. Summary of shared cell states and pathological features from cross-tissue, single-cell atlases of macrophages [74], T cells [75], and fibroblasts [76] in IMIDs. Black, straight arrows: cell state transitions; Black, curved arrows: cell taxis; Black arrows with molecules: cytokine release; Red arrows: pro-inflammatory effects. Created with BioRender.com.

There is a breadth of IMID tissue scRNA-seq data available from published studies. This includes RA synovium [69,70], psoriatic and dermatitis skin [71], colon, and ileum from IBD [72], kidney from lupus nephritis [73], and others. Despite this, relatively few cross-tissue, single-cell studies of IMIDs have yet been published. The three that have derive from the same group of investigators, each favouring the Harmony [34] data integration algorithm or variant thereof for purposes of data integration (Figure 2). First, a study by Zhang et al. [74] sought to identify shared immune compartments between COVID-19 bronchoalveolar lavage fluid (BALF) and inflamed tissue from IMIDs—namely, RA synovium, lupus nephritis kidney, CD ileum, and UC colon. Given differential cell type proportions between tissues, scRNA-seq data were integrated using a weighted variant of Harmony [34] to account for bias. Amongst the four inflammatory macrophage states identified, the authors described a CXCL10^+^CCL2^+^ “inflammatory” phenotype abundant in COVID-19 BALF that is also enriched in inflamed synovium, CD ileum, and UC colon compared to non-inflamed controls. Next, building on the observation that granzyme K-expressing CD8 T cells (termed tissue-enriched granzyme K-expressing or *T_teK_* CD8 cells) are abundant in the synovium of RA patients [69], Jonsson et al. [75] employed Harmony to integrate scSeq data from synovial tissue, gut, kidney, and COVID-19 BALF. They then confirmed *T_teK_* CD8 cells to be a major population of tissue-associated T cells across diseases and human tissues—being present at higher proportions than granzyme B-positive counterparts more classically associated with cytotoxicity. Indeed, *T_teK_* CD8 cells have a lower cytotoxic potential and are not exhausted, rather being prolific producers of interferon gamma (IFNγ) and TNF. Finally (also employing a weighted variant of Harmony [34] for integration), Korsunsky et al. [76] constructed an elegant fibroblast cell atlas with newly generated scRNA-seq data from ILD lung tissue, UC colon, salivary gland from primary Sjögren’s syndrome (pSS), and RA synovium. Amongst the five fibroblast states shared across tissues, the authors identified two—CXCL10^+^CCL19^+^ “immune-interacting” and SPARC^+^COL3A1^+^ “vascular-interacting” fibroblasts—to be expanded in all tissues. Respectively localised to lymphoid niches and perivascular regions of these inflamed tissues, the authors thereby proposed novel stromal drivers of immune cell infiltration and matrix remodelling common to distinct IMID tissues, with implications for therapeutic targeting.

These exemplars, transformative in their own right, emphasise the potential value of cross-tissue comparisons in IMIDs as a route to shared mechanistic understanding (Figure 2)—and additional important insights to be gained from replication by different researchers are awaited. They also invite important downstream mechanistic work as a precursor to potential interventional studies [44,46]. For example, Korsunsky et al. [76] went on to culture synovial fibroblasts from ILD and RA synovial tissue and, upon stimulating them with supernatant from in vitro-activated T cells, observed expansion of an immune-interacting fibroblast subtype reminiscent of that identified from scRNA-seq data. The vascular-interacting SPARC^+^COL3A1^+^ phenotype could only be recapitulated in a 3D synovial organoid model, which facilitates vascular tubule formation, as opposed to 2D co-culture with endothelial cells. This highlights the importance of culture conditions, including the importance of 3D architecture, when designing experiments to functionally validate scSeq findings in vitro as a route to better understanding IMID pathophysiology, and will inform future studies. 

The current paucity of cross-tissue, single-cell atlases of IMID should not be misdiagnosed as an absence of interest since large-scale projects are in development. For example, the Oxford-Janssen Cartography collaboration was launched in 2021 and aims to create detailed cellular atlases across multiple IMIDs to inform precision medicine. At this early stage, these researchers have developed a pipeline for multi-omic single-cell and spatial transcriptomic data analysis to facilitate this endeavour [77]. 

Importantly, many cell states that have been implicated in “tissue-discrete” immunopathologies have yet to be explored in a cross-tissue context. For example, T peripheral helper (Tph) cells were first characterised in seropositive RA synovium as a PD-1^hi^CXCR5^-^CD4^+^ population, which can infiltrate inflamed tissues and promote B cell maturation, thereby contributing to the formation of tertiary lymphoid structures [78]. Since this finding, Tph cells have been implicated in several autoimmune diseases, including systemic lupus erythematous (SLE) [79], type 1 diabetes (T1D) [80], primary biliary cirrhosis (PBC) [81], immunoglobin A nephropathy (IgAN) [82], and immunoglobin G4-related disease (IgG4-RD) [83]. Whilst these cells have displayed enrichment in individual diseases and correlation with disease activity, their cellular phenotypes have not been compared across tissues in integrative analyses. Indeed, beyond RA, Tph studies have been limited to circulating cells due to a lack of available tissue.

Reasons for a relative dearth of multi-tissue studies of IMIDs to date are, as touched on in the following section, various, and benchmarking of numerous available data integration algorithms in this specific context remains an area of unmet need. Nonetheless, these studies represent an excellent foundation and stimulus for future work.

## 5. Discussion

Advancements in single-cell genomics and the increasing availability of IMID scRNA-seq datasets have created great potential for multi-tissue, integrative analyses of IMIDs. The paucity of multi-tissue IMID atlases in part reflects challenges that extend beyond the field of immunobiology. Chief among these is data sharing, which is currently not standardised across institutions. Additional bureaucracy can lead to delays and possible omissions from integrative analyses, an outcome that is unfavourable for owners and prospective users of data alike. Indeed, the EU-STANDS4PM Consortium, a pan-European platform for standardisation in silico studies in personalised medicine, devised a harmonised data access agreement to achieve this very goal [84]. Another challenge for the integrative analysis of IMID tissue is that published scRNA-seq datasets are aligned to different versions (“builds”) of the human genome, a resource that is constantly evolving [85]. Consequently, access to raw sequencing reads in the form of FASTQ files is indispensable for integrative analyses. Despite this, some authors opt to publish processed data, while raw data are not always accessible for secondary analysis. As the advancement of new knowledge in immunobiology increasingly stands to benefit from large-scale integrative analyses of the kind described in this review, a remoulding of academic culture should be encouraged by institutions and publishers alike; this should remove barriers to data access whilst protecting intellectual ownership and appropriately crediting the originators of high-value translational data. Accordingly, we suggest it would be advantageous if scientific organisations, including the National Institute of Allergy and Infectious Diseases (NIAID) paid heed to this unmet need, for example, establishing consensus groups and open platforms for access to organised data and annotations. Further along in the integrative analysis pipeline, integration presents related challenges for multi-tissue atlases of IMID tissues. There are many effective algorithms for batch correction, but distinguishing batch effects from true biology and knowing when the latter has been removed are the challenges that remain. To mitigate this, multi-tissue atlases should be supplemented with orthogonal validation of findings using methods such as spatial profiling, immunohistochemistry and in vitro studies.

Multi-tissue atlases have enormous potential to address gaps in our understanding of cell states in inflammatory diseases. Cross-tissue studies of IMIDs have demonstrated that previously identified inflammatory cell states in one tissue can be resolved in multiple other tissues via integration [74,75,76]. Another knowledge gap is linked to this: cell–cell interactions in the inflammatory microenvironment are understudied in comparison to the characterisation of cell states within individual cell types. Spatial transcriptomics, in combination with cell–cell interaction prediction software, can be leveraged to bridge this gap, similar to pan-cancer studies [63,67]. Multi-tissue atlases must also address knowledge gaps at the disease level. Common IMIDs such as RA, CD, and UC regularly feature in meta- and integrative analyses of inflamed tissue, leading to other diseases being overlooked. For example, giant cell arteritis (GCA), the most common form of vasculitis [86], is rarely included in these analyses. A diversification of IMID tissues in integrative analyses would aid in the identification of rare cell populations that could be shared across them. The underlying hypothesis in these studies is that there are a finite number of cell states and transcriptional programmes that define responses to different stimuli, rather than the possibility that each inflammatory challenge induces a distinct response. There is some encouraging evidence for this, such as circulating Tph cells across IMIDs [87] and shared fibroblast states across inflamed tissues [76], but a comprehensive catalogue of these states is lacking. Defining these states has the potential to clarify shared qualities across IMIDs. Therefore, integrative analysis of tissues from rare and common IMID tissues provides an opportunity for cross-fertilisation between cohorts with different diseases, benefitting both patient groups. Finally, multi-tissue atlases must help address the lack of association between shared cell states and therapeutic outcomes. The association of shared cell states with favourable and unfavourable responses to immunotherapy would aid patient stratification, improving the efficacy of these interventions. This will require bulk and/or scRNA-seq datasets associated with phase II/III clinical trials for targeted immunotherapies with high-quality metadata. Collaboration between industry and academia will be vital in this endeavour, with well-curated clinical trial samples at their strategic centre.

In conclusion, multi-tissue atlases have charted shared and tissue-discrete biology in health and cancer; IMIDs are now beginning to be examined in the same manner. Although currently limited in number, these studies’ obvious potential will lead to inevitable growth in the coming years. We propose they will ultimately illuminate novel IMID taxonomies, heralding a new era of precision medicine for patients.

## Figures and Tables

**Table 1:** Examples of methods for integration of scRNA-seq datasets. Adapted from [25].

	Language	Dimension Reduction	Similarity Search Level	Output Type (G/E/W) *	Notes	Reference
MNN	R	-	Cell	G		[26]
fastMNN	R	PCA	Cell	G	Good for simple integration tasks [27]	[26]
Seurat v2 (CCA)	R	CCA	Cell	E		[28]
Seurat v3	R	CCA	Cell	G	High usability [27]	[29]
Scanorama	Python	SVD	Cell	G/E	Good for simple integration tasks [27]	[30]
BBKNN	Python	PCA	Cell	W	High speed and usability [27]	[31]
Conos	R	PCA	Cell	W		[32]
Harmony	R	PCA	Cluster	E	Good for simple integration tasks. High speed and usability [27,33]	[34]
LIGER	R	iNMF	Cluster	E		[35]
scMerge	R	PCA	Cluster	G		[36]
scVI	Python	VAE	-	E	Good for complex integration tasks. Memory efficient [27]	[37]
scANVI	Python	VAE	-	E	Good for complex integration tasks. Memory efficient. Requires cell annotations [27]	[38]
scGen	Python	VAE	-	G	Requires cell annotations	[39]
trVAE	Python	VAE	-	E		[40]

MNN, mutual nearest neighbours; PCA, principal component analysis; CCA, canonical correlation analysis; SVD, single value decomposition; iNMF, integrative non-negative matrix factorisation; VAE, variational autoencoder. * G, gene expression matrix; E, embeddings; W, weighted edge graph.

**Table 2:** Examples of benchmarking metrics for integration of scRNA-seq datasets. Adapted from [27,41].

	Metric Name	Level	Notes	Reference
Batch mixing	iLISI	Cell	Inverse of the sum of batch probabilities within a weighted kNN. Reflects the number of batches in a neighbourhood. Graph variant scales to large datasets	[27,34]
	kBET	Cell type	Comparison of label composition of a k-nearest neighbourhood of a cell and the expected (global) label composition	[42]
	Graph connectivity	Cell type	Determines how well the kNN graph of the integrated data connects cells of the same label	[27]
	ASW batch	Cell	Relationship between within-batch and between batch distances of a cell. Reflects separation between batches	[43]
	PCR batch	Global	Correlation of batch variable with principal components weighted by variance contribution. Reflects the total variance explained by the batch variable	[42]
Bio-conservation	cLISI	Cell	Inverse of the sum of cell type probabilities within a weighted kNN. Reflects the number of cell types in a neighbourhood. Graph variant scales to large datasets	[27,34]
	ASW label	Cell type	Relationship between within-label and between-label distances of a cell. Reflects separation between cell type clusters	[43]
	Isolated label	Cell type	Determines how well cell type labels that are shared by few batches are separated from other cell type labels	[27]
	KMeans NMI	Cell type	Overlap between predicted clustering and provided cell type labels	[44]
	KMeans ARI	Cell type	Overlap between predicted clustering and provided cell type labels (after correcting for overlap by chance)	[45]

iLISI, integration local inverse Simpson’s index; kNN, k-nearest neighbourhood; kBET, k-nearest-neighbour batch effect test; ASW, average silhouette width; PCR, principal component regression; cLISI, cell-type local inverse Simpson’s index; NMI, normalised mutual information; ARI, adjusted rand index.

**Table 3:** Examples of cell type annotation methods of scRNA-seq datasets. Adapted from [46,47].

	Method Name	Language	Approach	Reference
Marker-based	scCATCH	R	Scoring system	[48]
	SCSA	R	Scoring system	[49]
	SCINA	Python	Bi-modal distribution fit to marker genes	[50]
	CellAssign	R	Probabilistic Bayesian model	[51]
Reference-based	scmap-cell	R	Cosine similarity	[52]
	scmap-cluster	R	Cosine similarity, Pearson/Spearman correlation	[52]
	SingleR	R	Spearman correlation	[53]
	scMatch	Python	Spearman correlation	[54]
	CHETAH	R	Spearman correlation	[55]
	CellTypist	Python	Logistic regression classifier	[14]
	scPred	R	SVM *	[56]
	SingleCellNet	R	Random forest	[57]
	scNym	Python	Adversarial neural network	[58]
	Seurat (Azimuth)	R	Reference mapping + Transfer learning	[59]
	scArches	Python	Reference mapping + Transfer learning	[60]
	Symphony	R	Reference mapping + Transfer learning	[61]

* SVM, support vector machine.

## Data Availability

Not applicable.

## References

[1] Monteleone G., Moscardelli A., Colella A., Marafini I., Salvatori S. (2023). Immune-Mediated Inflammatory Diseases: Common and Different Pathogenic and Clinical Features. Autoimmun. Rev..

[2] Conrad N., Misra S., Verbakel J.Y., Verbeke G., Molenberghs G., Taylor P.N., Mason J., Sattar N., McMurray J.J.V., McInnes I.B. (2023). Incidence, Prevalence, and Co-Occurrence of Autoimmune Disorders over Time and by Age, Sex, and Socioeconomic Status: A Population-Based Cohort Study of 22 Million Individuals in the UK. Lancet.

[3] Monaco C., Nanchahal J., Taylor P., Feldmann M. (2015). Anti-TNF Therapy: Past, Present and Future. Int. Immunol..

[4] Landewé R., Braun J., Deodhar A., Dougados M., Maksymowych W.P., Mease P.J., Reveille J.D., Rudwaleit M., van der Heijde D., Stach C. (2014). Efficacy of Certolizumab Pegol on Signs and Symptoms of Axial Spondyloarthritis Including Ankylosing Spondylitis: 24-Week Results of a Double-Blind Randomised Placebo-Controlled Phase 3 Study. Ann. Rheum. Dis..

[5] Torres J., Mehandru S., Colombel J.-F., Peyrin-Biroulet L. (2017). Crohn’s Disease. Lancet.

[6] Ungaro R., Mehandru S., Allen P.B., Peyrin-Biroulet L., Colombel J.-F. (2017). Ulcerative Colitis. Lancet.

[7] Buckley C.D., Chernajovsky L., Chernajovsky Y., Modis L.K., O’Neill L.A., Brown D., Connor R., Coutts D., Waterman E.A., Tak P.P. (2021). Immune-Mediated Inflammation across Disease Boundaries: Breaking down Research Silos. Nat Immunol.

[8] Clark A.D., Nair N., Anderson A.E., Thalayasingam N., Naamane N., Skelton A.J., Diboll J., Barton A., Eyre S., Isaacs J.D. (2020). Lymphocyte DNA Methylation Mediates Genetic Risk at Shared Immune-Mediated Disease Loci. J. Allergy Clin. Immunol..

[9] Papalexi E., Satija R. (2018). Single-Cell RNA Sequencing to Explore Immune Cell Heterogeneity. Nat. Rev. Immunol..

[10] Hao Y., Stuart T., Kowalski M.H., Choudhary S., Hoffman P., Hartman A., Srivastava A., Molla G., Madad S., Fernandez-Granda C. (2024). Dictionary Learning for Integrative, Multimodal and Scalable Single-Cell Analysis. Nat. Biotechnol..

[11] Wolf F.A., Angerer P., Theis F.J. (2018). SCANPY: Large-Scale Single-Cell Gene Expression Data Analysis. Genome Biol..

[12] Regev A., Teichmann S.A., Lander E.S., Amit I., Benoist C., Birney E., Bodenmiller B., Campbell P., Carninci P., Clatworthy M. (2017). The Human Cell Atlas. eLife.

[13] Lindeboom R.G.H., Regev A., Teichmann S.A. (2021). Towards a Human Cell Atlas: Taking Notes from the Past. Trends Genet..

[14] Domínguez Conde C., Xu C., Jarvis L.B., Rainbow D.B., Wells S.B., Gomes T., Howlett S.K., Suchanek O., Polanski K., King H.W. (2022). Cross-Tissue Immune Cell Analysis Reveals Tissue-Specific Features in Humans. Science.

[15] Suo C., Dann E., Goh I., Jardine L., Kleshchevnikov V., Park J.-E., Botting R.A., Stephenson E., Engelbert J., Tuong Z.K. (2022). Mapping the Developing Human Immune System across Organs. Science.

[16] Eraslan G., Drokhlyansky E., Anand S., Fiskin E., Subramanian A., Slyper M., Wang J., Van Wittenberghe N., Rouhana J.M., Waldman J. (2022). Single-Nucleus Cross-Tissue Molecular Reference Maps toward Understanding Disease Gene Function. Science.

[17] The Tabula Sapiens Consortium (2022). The Tabula Sapiens: A Multiple-Organ, Single-Cell Transcriptomic Atlas of Humans. Science.

[18] Donlin L.T., Rao D.A., Wei K., Slowikowski K., McGeachy M.J., Turner J.D., Meednu N., Mizoguchi F., Gutierrez-Arcelus M., Lieb D.J. (2018). Methods for High-Dimensional Analysis of Cells Dissociated from Cryopreserved Synovial Tissue. Arthritis Res. Ther..

[19] Zheng G.X.Y., Terry J.M., Belgrader P., Ryvkin P., Bent Z.W., Wilson R., Ziraldo S.B., Wheeler T.D., McDermott G.P., Zhu J. (2017). Massively Parallel Digital Transcriptional Profiling of Single Cells. Nat. Commun..

[20] Ortolano N. The neXt Generation of Single Cell RNA-Seq: An Introduction to GEM-X Technology. 10x Genomics. https://www.10xgenomics.com/blog/the-next-generation-of-single-cell-rna-seq-an-introduction-to-gem-x-technology.

[21] Miga K.H., Newton Y., Jain M., Altemose N., Willard H.F., Kent W.J. (2014). Centromere Reference Models for Human Chromosomes X and Y Satellite Arrays. Genome Res..

[22] Martin F.J., Amode M.R., Aneja A., Austine-Orimoloye O., Azov A.G., Barnes I., Becker A., Bennett R., Berry A., Bhai J. (2023). Ensembl 2023. Nucleic Acids Res..

[23] Freeberg M.A., Fromont L.A., D’Altri T., Romero A.F., Ciges J.I., Jene A., Kerry G., Moldes M., Ariosa R., Bahena S. (2022). The European Genome-Phenome Archive in 2021. Nucleic Acids Res..

[24] Barrett T., Wilhite S.E., Ledoux P., Evangelista C., Kim I.F., Tomashevsky M., Marshall K.A., Phillippy K.H., Sherman P.M., Holko M. (2013). NCBI GEO: Archive for Functional Genomics Data Sets—Update. Nucleic Acids Res..

[25] Ryu Y., Han G.H., Jung E., Hwang D. (2023). Integration of Single-Cell RNA-Seq Datasets: A Review of Computational Methods. Mol. Cells.

[26] Haghverdi L., Lun A.T.L., Morgan M.D., Marioni J.C. (2018). Batch Effects in Single-Cell RNA-Sequencing Data Are Corrected by Matching Mutual Nearest Neighbors. Nat. Biotechnol..

[27] Luecken M.D., Büttner M., Chaichoompu K., Danese A., Interlandi M., Mueller M.F., Strobl D.C., Zappia L., Dugas M., Colomé-Tatché M. (2022). Benchmarking Atlas-Level Data Integration in Single-Cell Genomics. Nat. Methods.

[28] Butler A., Hoffman P., Smibert P., Papalexi E., Satija R. (2018). Integrating Single-Cell Transcriptomic Data across Different Conditions, Technologies, and Species. Nat. Biotechnol..

[29] Stuart T., Butler A., Hoffman P., Hafemeister C., Papalexi E., Mauck W.M., Hao Y., Stoeckius M., Smibert P., Satija R. (2019). Comprehensive Integration of Single-Cell Data. Cell.

[30] Hie B., Bryson B., Berger B. (2019). Efficient Integration of Heterogeneous Single-Cell Transcriptomes Using Scanorama. Nat. Biotechnol..

[31] Polański K., Young M.D., Miao Z., Meyer K.B., Teichmann S.A., Park J.-E. (2020). BBKNN: Fast Batch Alignment of Single Cell Transcriptomes. Bioinformatics.

[32] Barkas N., Petukhov V., Nikolaeva D., Lozinsky Y., Demharter S., Khodosevich K., Kharchenko P.V. (2019). Joint Analysis of Heterogeneous Single-Cell RNA-Seq Dataset Collections. Nat. Methods.

[33] Tran H.T.N., Ang K.S., Chevrier M., Zhang X., Lee N.Y.S., Goh M., Chen J. (2020). A Benchmark of Batch-Effect Correction Methods for Single-Cell RNA Sequencing Data. Genome Biol..

[34] Korsunsky I., Millard N., Fan J., Slowikowski K., Zhang F., Wei K., Baglaenko Y., Brenner M., Loh P., Raychaudhuri S. (2019). Fast, Sensitive and Accurate Integration of Single-Cell Data with Harmony. Nat. Methods.

[35] Welch J.D., Kozareva V., Ferreira A., Vanderburg C., Martin C., Macosko E.Z. (2019). Single-Cell Multi-Omic Integration Compares and Contrasts Features of Brain Cell Identity. Cell.

[36] Lin Y., Ghazanfar S., Wang K.Y.X., Gagnon-Bartsch J.A., Lo K.K., Su X., Han Z.-G., Ormerod J.T., Speed T.P., Yang P. (2019). scMerge Leverages Factor Analysis, Stable Expression, and Pseudoreplication to Merge Multiple Single-Cell RNA-Seq Datasets. Proc. Natl. Acad. Sci. USA.

[37] Lopez R., Regier J., Cole M.B., Jordan M.I., Yosef N. (2018). Deep Generative Modeling for Single-Cell Transcriptomics. Nat. Methods.

[38] Xu C., Lopez R., Mehlman E., Regier J., Jordan M.I., Yosef N. (2021). Probabilistic Harmonization and Annotation of Single-cell Transcriptomics Data with Deep Generative Models. Mol. Syst. Biol..

[39] Lotfollahi M., Wolf F.A., Theis F.J. (2019). scGen Predicts Single-Cell Perturbation Responses. Nat. Methods.

[40] Lotfollahi M., Naghipourfar M., Theis F.J., Wolf F.A. (2020). Conditional Out-of-Distribution Generation for Unpaired Data Using Transfer VAE. Bioinformatics.

[41] Lütge A., Zyprych-Walczak J., Kunzmann U.B., Crowell H.L., Calini D., Malhotra D., Soneson C., Robinson M.D. (2021). CellMixS: Quantifying and Visualizing Batch Effects in Single-Cell RNA-Seq Data. Life Sci. Alliance.

[42] Büttner M., Miao Z., Wolf F.A., Teichmann S.A., Theis F.J. (2019). A Test Metric for Assessing Single-Cell RNA-Seq Batch Correction. Nat. Methods.

[43] Rousseeuw P.J. (1987). Silhouettes: A Graphical Aid to the Interpretation and Validation of Cluster Analysis. J. Comput. Appl. Math..

[44] Pedregosa F., Varoquaux G., Gramfort A., Michel V., Thirion B., Grisel O., Blondel M., Prettenhofer P., Weiss R., Dubourg V. (2011). Scikit-Learn: Machine Learning in Python. J. Mach. Learn. Res..

[45] Hubert L., Arabie P. (1985). Comparing Partitions. J. Classif..

[46] Pasquini G., Arias J.E.R., Schäfer P., Busskamp V. (2021). Automated Methods for Cell Type Annotation on scRNA-Seq Data. Comput. Struct. Biotechnol. J..

[47] Xie B., Jiang Q., Mora A., Li X. (2021). Automatic Cell Type Identification Methods for Single-Cell RNA Sequencing. Comput. Struct. Biotechnol. J..

[48] Shao X., Liao J., Lu X., Xue R., Ai N., Fan X. (2020). scCATCH: Automatic Annotation on Cell Types of Clusters from Single-Cell RNA Sequencing Data. iScience.

[49] Cao Y., Wang X., Peng G. (2020). SCSA: A Cell Type Annotation Tool for Single-Cell RNA-Seq Data. Front. Genet..

[50] Zhang Z., Luo D., Zhong X., Choi J.H., Ma Y., Wang S., Mahrt E., Guo W., Stawiski E.W., Modrusan Z. (2019). SCINA: A Semi-Supervised Subtyping Algorithm of Single Cells and Bulk Samples. Genes.

[51] Zhang A.W., O’Flanagan C., Chavez E.A., Lim J.L.P., Ceglia N., McPherson A., Wiens M., Walters P., Chan T., Hewitson B. (2019). Probabilistic Cell-Type Assignment of Single-Cell RNA-Seq for Tumor Microenvironment Profiling. Nat. Methods.

[52] Kiselev V.Y., Yiu A., Hemberg M. (2018). Scmap: Projection of Single-Cell RNA-Seq Data across Data Sets. Nat. Methods.

[53] Aran D., Looney A.P., Liu L., Wu E., Fong V., Hsu A., Chak S., Naikawadi R.P., Wolters P.J., Abate A.R. (2019). Reference-Based Analysis of Lung Single-Cell Sequencing Reveals a Transitional Profibrotic Macrophage. Nat. Immunol..

[54] Hou R., Denisenko E., Forrest A.R.R. (2019). scMatch: A Single-Cell Gene Expression Profile Annotation Tool Using Reference Datasets. Bioinformatics.

[55] de Kanter J.K., Lijnzaad P., Candelli T., Margaritis T., Holstege F.C.P. (2019). CHETAH: A Selective, Hierarchical Cell Type Identification Method for Single-Cell RNA Sequencing. Nucleic Acids Res..

[56] Alquicira-Hernandez J., Sathe A., Ji H.P., Nguyen Q., Powell J.E. (2019). scPred: Accurate Supervised Method for Cell-Type Classification from Single-Cell RNA-Seq Data. Genome Biol..

[57] Tan Y., Cahan P. (2019). SingleCellNet: A Computational Tool to Classify Single Cell RNA-Seq Data Across Platforms and Across Species. Cell Syst..

[58] Kimmel J.C., Kelley D.R. (2021). Semisupervised Adversarial Neural Networks for Single-Cell Classification. Genome Res..

[59] Hao Y., Hao S., Andersen-Nissen E., Mauck W.M., Zheng S., Butler A., Lee M.J., Wilk A.J., Darby C., Zager M. (2021). Integrated Analysis of Multimodal Single-Cell Data. Cell.

[60] Lotfollahi M., Naghipourfar M., Luecken M.D., Khajavi M., Büttner M., Wagenstetter M., Avsec Ž., Gayoso A., Yosef N., Interlandi M. (2022). Mapping Single-Cell Data to Reference Atlases by Transfer Learning. Nat. Biotechnol..

[61] Kang J.B., Nathan A., Weinand K., Zhang F., Millard N., Rumker L., Moody D.B., Korsunsky I., Raychaudhuri S. (2021). Efficient and Precise Single-Cell Reference Atlas Mapping with Symphony. Nat. Commun..

[62] Zheng L., Qin S., Si W., Wang A., Xing B., Gao R., Ren X., Wang L., Wu X., Zhang J. (2021). Pan-Cancer Single-Cell Landscape of Tumor-Infiltrating T Cells. Science.

[63] Chu Y., Dai E., Li Y., Han G., Pei G., Ingram D.R., Thakkar K., Qin J.-J., Dang M., Le X. (2023). Pan-Cancer T Cell Atlas Links a Cellular Stress Response State to Immunotherapy Resistance. Nat. Med..

[64] Tang F., Li J., Qi L., Liu D., Bo Y., Qin S., Miao Y., Yu K., Hou W., Li J. (2023). A Pan-Cancer Single-Cell Panorama of Human Natural Killer Cells. Cell.

[65] Efremova M., Vento-Tormo M., Teichmann S.A., Vento-Tormo R. (2020). CellPhoneDB: Inferring Cell–Cell Communication from Combined Expression of Multi-Subunit Ligand–Receptor Complexes. Nat. Protoc..

[66] Cheng S., Li Z., Gao R., Xing B., Gao Y., Yang Y., Qin S., Zhang L., Ouyang H., Du P. (2021). A Pan-Cancer Single-Cell Transcriptional Atlas of Tumor Infiltrating Myeloid Cells. Cell.

[67] Ma C., Yang C., Peng A., Sun T., Ji X., Mi J., Wei L., Shen S., Feng Q. (2023). Pan-Cancer Spatially Resolved Single-Cell Analysis Reveals the Crosstalk between Cancer-Associated Fibroblasts and Tumor Microenvironment. Mol. Cancer.

[68] Street K., Risso D., Fletcher R.B., Das D., Ngai J., Yosef N., Purdom E., Dudoit S. (2018). Slingshot: Cell Lineage and Pseudotime Inference for Single-Cell Transcriptomics. BMC Genom..

[69] Zhang F., Wei K., Slowikowski K., Fonseka C.Y., Rao D.A., Kelly S., Goodman S.M., Tabechian D., Hughes L.B., Salomon-Escoto K. (2019). Defining Inflammatory Cell States in Rheumatoid Arthritis Joint Synovial Tissues by Integrating Single-Cell Transcriptomics and Mass Cytometry. Nat. Immunol..

[70] Zhang F., Jonsson A.H., Nathan A., Millard N., Curtis M., Xiao Q., Gutierrez-Arcelus M., Apruzzese W., Watts G.F.M., Weisenfeld D. (2023). Deconstruction of Rheumatoid Arthritis Synovium Defines Inflammatory Subtypes. Nature.

[71] Reynolds G., Vegh P., Fletcher J., Poyner E.F.M., Stephenson E., Goh I., Botting R.A., Huang N., Olabi B., Dubois A. (2021). Developmental Cell Programs Are Co-Opted in Inflammatory Skin Disease. Science.

[72] Kong L., Pokatayev V., Lefkovith A., Carter G.T., Creasey E.A., Krishna C., Subramanian S., Kochar B., Ashenberg O., Lau H. (2023). The Landscape of Immune Dysregulation in Crohn’s Disease Revealed through Single-Cell Transcriptomic Profiling in the Ileum and Colon. Immunity.

[73] Arazi A., Rao D.A., Berthier C.C., Davidson A., Liu Y., Hoover P.J., Chicoine A., Eisenhaure T.M., Jonsson A.H., Li S. (2019). The Immune Cell Landscape in Kidneys of Patients with Lupus Nephritis. Nat. Immunol..

[74] Zhang F., Mears J.R., Shakib L., Beynor J.I., Shanaj S., Korsunsky I., Nathan A., Donlin L.T., Raychaudhuri S., Accelerating Medicines Partnership Rheumatoid Arthritis and Systemic Lupus Erythematosus (AMP RA/SLE) Consortium (2021). IFN-γ and TNF-α Drive a CXCL10+ CCL2+ Macrophage Phenotype Expanded in Severe COVID-19 Lungs and Inflammatory Diseases with Tissue Inflammation. Genome Med..

[75] Jonsson A.H., Zhang F., Dunlap G., Gomez-Rivas E., Watts G.F.M., Faust H.J., Rupani K.V., Mears J.R., Meednu N., Wang R. (2022). Granzyme K+ CD8 T Cells Form a Core Population in Inflamed Human Tissue. Sci. Transl. Med..

[76] Korsunsky I., Wei K., Pohin M., Kim E.Y., Barone F., Major T., Taylor E., Ravindran R., Kemble S., Watts G.F.M. (2022). Cross-Tissue, Single-Cell Stromal Atlas Identifies Shared Pathological Fibroblast Phenotypes in Four Chronic Inflammatory Diseases. Med.

[77] Curion F., Rich-Griffin C., Agarwal D., Ouologuem S., Thomas T., Theis F.J., Dendrou C.A. (2023). Panpipes: A Pipeline for Multiomic Single-Cell and Spatial Transcriptomic Data Analysis. bioRxiv.

[78] Rao D.A., Gurish M.F., Marshall J.L., Slowikowski K., Fonseka C.Y., Liu Y., Donlin L.T., Henderson L.A., Wei K., Mizoguchi F. (2017). Pathologically Expanded Peripheral T Helper Cell Subset Drives B Cells in Rheumatoid Arthritis. Nature.

[79] Bocharnikov A.V., Keegan J., Wacleche V.S., Cao Y., Fonseka C.Y., Wang G., Muise E.S., Zhang K.X., Arazi A., Keras G. (2019). PD-1^hi^CXCR5^–^ T Peripheral Helper Cells Promote B Cell Responses in Lupus via MAF and IL-21. JCI Insight.

[80] Ekman I., Ihantola E.-L., Viisanen T., Rao D.A., Näntö-Salonen K., Knip M., Veijola R., Toppari J., Ilonen J., Kinnunen T. (2019). Circulating CXCR5−PD-1hi Peripheral T Helper Cells Are Associated with Progression to Type 1 Diabetes. Diabetologia.

[81] Yong L., Chunyan W., Yan Y., Wanyu L., Huifan J., Pingwei Z., Yanfang J. (2021). Expanded Circulating Peripheral Helper T Cells in Primary Biliary Cholangitis: Tph Cells in PBC. Mol. Immunol..

[82] Wang X., Li T., Si R., Chen J., Qu Z., Jiang Y. (2020). Increased Frequency of PD-1hiCXCR5- T Cells and B Cells in Patients with Newly Diagnosed IgA Nephropathy. Sci. Rep..

[83] Zhang P., Wang M., Chen Y., Li J., Liu Z., Lu H., Fei Y., Feng R., Zhao Y., Zeng X. (2022). Expanded CD4+CXCR5-PD-1+ Peripheral T Helper like Cells and Clinical Significance in IgG4-Related Disease. Clin. Immunol..

[84] EU-STANDS4PM Harmonised Data Access Agreement (hDAA) for Sharing and Using Controlled Access Data. https://www.eu-stands4pm.eu/data_access.

[85] Schneider V.A., Graves-Lindsay T., Howe K., Bouk N., Chen H.-C., Kitts P.A., Murphy T.D., Pruitt K.D., Thibaud-Nissen F., Albracht D. (2017). Evaluation of GRCh38 and de Novo Haploid Genome Assemblies Demonstrates the Enduring Quality of the Reference Assembly. Genome Res..

[86] Sharma A., Mohammad A.J., Turesson C. (2020). Incidence and Prevalence of Giant Cell Arteritis and Polymyalgia Rheumatica: A Systematic Literature Review. Semin. Arthritis Rheum..

[87] Zou X., Huo F., Sun L., Huang J. (2024). Peripheral Helper T Cells in Human Diseases. J. Autoimmun..

